# EU-funded malaria research under the 6^th ^and 7^th ^Framework Programmes for research and technological development

**DOI:** 10.1186/1475-2875-10-11

**Published:** 2011-01-14

**Authors:** Andreas Holtel, Marita Troye-Blomberg, Inmaculada Penas-Jimenez

**Affiliations:** 1 Health Directorate, Directorate General for Research and Innovation, European Commission, Rue du Champ de Mars 21, 1050 Brussels, Belgium; 2 Department of Immunology, Wenner-Gren Institute, Stockholm University, Svante Arrheniusväg 16, SE 106 91 Stockholm, Sweden

## Abstract

While malaria research has traditionally been strong in Europe, targeted and sustained support for cooperative malaria research at EU level, namely through the EU's 6th and 7th Framework Programmes for research and technological development, FP6 (2002-2006) and FP7 (2007-2013), has boosted both impact and visibility of European malaria research. Most of the European malaria research community is now organized under a number of comprehensive and complementary research networks and projects, assembled around four key areas: (1) fundamental research on the malaria parasite and the disease, (2) development of new malaria drugs, (3) research and development of a malaria vaccine, and (4) research to control the malaria-transmitting mosquito vector. Considerable efforts were undertaken to ensure adequate participation of research groups from disease-endemic countries, in particular from Africa, with the long-term aim to strengthen cooperative links and research capacities in these countries. The concept of organizing European research through major strategic projects to form a "European Research Area" (ERA) was originally developed in the preparation of FP6, and ERA formation has now turned into a major EU policy objective explicitly inscribed into the Lisbon Treaty. EU-funded malaria research may serve as a showcase to demonstrate how ERA formation can successfully be implemented in a given area of science when several surrounding parameters converge to support implementation of this strategic concept: timely coincidence of political stimuli, responsive programming, a clearly defined - and well confined - area of research, and the readiness of the targeted research community who is well familiar with transnational cooperation at EU level. Major EU-funded malaria projects have evolved into thematic and organizational platforms that can collaborate with other global players. Europe may thus contribute more, and better, to addressing the global research agenda for malaria.

## The global burden of malaria

According to the World Health Organization (WHO), there were in 2008 an estimated 247 million malaria cases among more than 3 billion people at risk, causing nearly one million deaths (even much more according to other estimates), mostly of children under 5 years and pregnant women [1,2]. Malaria is a vector-borne disease transmitted to humans through the bite of female Anopheles mosquitoes. The most lethal of the species of malaria parasites that infect humans is *Plasmodium falciparum*, which is common in sub-Saharan Africa but also present in tropical and subtropical areas of Central and South America, Asia and in the Middle East [2]. Analysis of the relationship between malaria burden and socio-economic factors reveals that the global distribution of malaria shows a striking correlation with the respective per-capita gross domestic product (GPD), with lower rates of economic growth in malaria-endemic countries [3]. In light of the United Nations' Millenium Development goals, global malaria control efforts have been challenged by the Bill and Melinda Gates Foundation (BMGF) together with WHO's Global Malaria Action Plan (GMAP), aiming to totally eradicate malaria, or as a short-term goal, to eliminate malaria on a country-by-country basis [4].

## Malaria research in Europe

Malaria research in Europe has always been strong, historically being driven by the need to cope with tropical diseases encountered in the former colonies of European nations [5-7]. Furthermore, until eradication campaigns were successfully conducted in the first half of the 20th century, malaria was endemic in large parts of southern Europe, notably in Italy (where the name " mal aire" was coined to designate the disease), but also in southern France, Greece and the whole of the Balkan region [8,9]. Typically, malaria research in Europe was, and to a large extent still is, conducted either in national research institutions dedicated to tropical medicine (e.g. Schools of, or Institutes for, Tropical Medicine, Hygiene, Parasitology etc), or in a scatter of specialized university hospitals where tropical diseases had become a major focus of clinical research. Former European colonial powers also set up dedicated clinical research institutions located in their former malaria-endemic colonies, e.g. the British MRC Unit in The Gambia or the, originally French, Institut Pasteur in Senegal. In addition, a number of both small and larger European pharmaceutical companies have continuously been active in the area of malaria drugs and vaccines research [10].

## European Union Funding for Malaria Research: from FP5 to FP7

Building on European Member States' national investments in malaria research and reflecting the EU's political commitment to contribute to the global development goals from research side [11], malaria research has received rather sustained funding under the EU's recent research framework programmes. In the 5^th ^Framework Programme (FP5, 1998-2002), under the "International Co-operation (INCO)" and the "Control of Infectious Diseases" sections, around € 35 million were spent on a total of ~ 30 separate malaria co-operation projects (with a mean volume of around € 1 million per project) [12]. A high-level round table conference on major communicable diseases in Brussels stressed again in the year 2000 the importance of research to combat the three big killer diseases. The EU responded to this political call in 2001 by setting up the first comprehensive "Programme of Action: accelerated action on HIV/AIDS, malaria and tuberculosis in the context of poverty reduction" [11], which comprised a strong section on research support. This coincided with the new structural approach proposed by the European Commission for the 6^th ^Framework Programme (FP6, 2002-2006), aimed at forming a rationally structured, open and sustained "European Research Area" (ERA) [13]. In this context, not only were some of the thematic areas re-examined for more targeted EU research support, but also the funding tools to address these research areas under FP6 were revised to become larger and more structurally organized projects [14]. In the course of these processes, the "Three Big Poverty-related Diseases" (HIV/AIDS, malaria and tuberculosis) acquired a clearly defined thematic, organizational and budgetary focus under FP6, a novelty under the EU research framework programmes. The ambition under FP6 was to overcome the fragmentation of important areas of research in Europe and to move towards a more structured, but open "European Research Area" (ERA). In practical terms, FP6 was designed to address key thematic areas through strategically placed, large-scale European research projects and networks, assembled around major scientific objectives and aiming to structure the respective field of research. The currently on-going FP7 programme builds in essence on the FP6 concept, still placing emphasis on large, high impact, and/or ERA-structuring projects [15]. A research focus on HIV/AIDS, malaria and tuberculosis, has been maintained also under FP7.

## Implementing the European Research Area (ERA) in the field of malaria research

A consultation of the science community, held at the beginning of FP6, identified topical research areas for FP6 as well as appropriate funding schemes to address them. In the case of malaria research, this consultation yielded very straightforward and clear recommendations:

(1) Fundamental malaria researchers should organize themselves in a large and comprehensive "Network of Excellence";

(2) European efforts to discover and develop new anti-malarial medicines should be grouped and managed under the umbrella of an "Integrated Project" for new malaria drugs; and

(3) European research groups aiming to prevent malaria by developing a vaccine should organize themselves under a larger "Integrated Project" on malaria vaccines.

**Figure 1:**
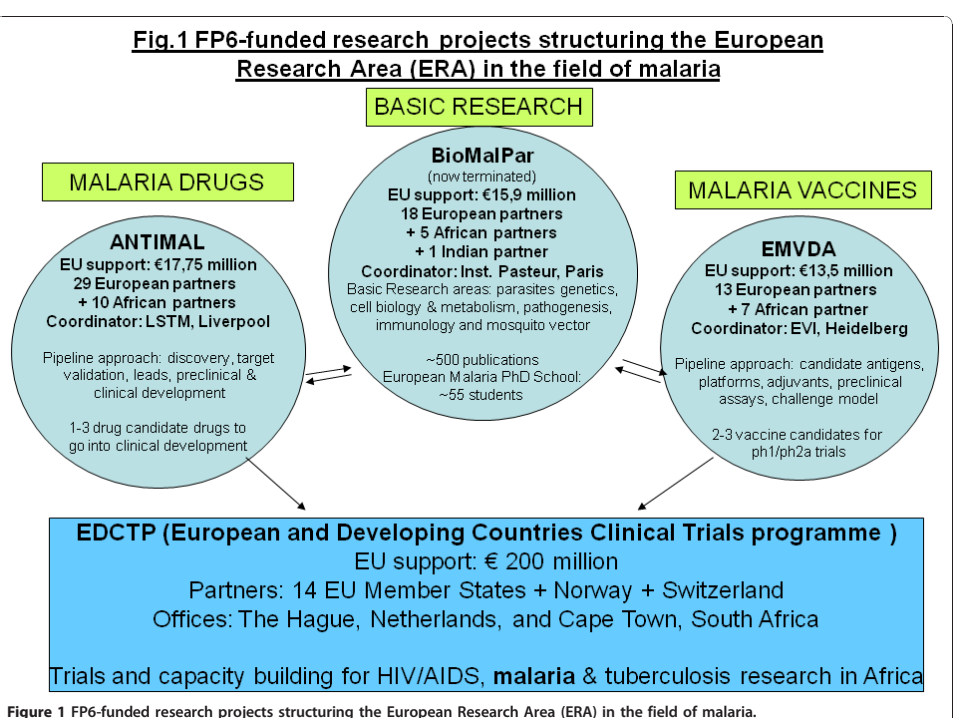
FP6-funded research projects structuring the European Research Area (ERA) in the field of malaria

Funding for Integrated Projects and Networks of Excellence under FP6 normally ranged between € 10 and 20 million each, which constituted a significant rise in project funding as compared with previous framework programmes, but which was still insufficient to allow for clinical testing of potential new drugs or vaccines in disease-endemic settings, principally in Africa. Thus, in order to provide EU support also for the costly and logistically demanding clinical testing part of the product development pipeline, namely for phase 2 and phase 3 clinical efficacy studies in Africa, a major new initiative, sourced by € 200 million of FP6 funds and matching investments from Member States, was launched in 2003, the "European and Developing Countries Clinical Trials Partnership (EDCTP) [16]. The remit of EDCTP is to test new interventions to confront HIV/AIDS, malaria and tuberculosis in clinical trials in Africa, and here a significant proportion of EDCTP funds goes towards testing new drugs and vaccines for malaria, next to new interventions for HIV and tuberculosis. Figure 1 depicts how the strategic recommendations for malaria research at the start of FP6 between 2003 and 2006 were translated into corresponding EU-funded cooperative networks.

(1) "BioMalPar", a Network of Excellence coordinated by Institut Pasteur in Paris, conducting fundamental research on "Biology and pathology of the malaria parasite" [17];

(2) "ANTIMAL", an Integrated Project coordinated by the Liverpool School of Tropical Medicine, on the "Development of New Drugs for the Treatment of Malaria" [18], and

(3) "EMVDA", i.e. the "European Malaria Vaccine Development Association" [19], an Integrated Project originally coordinated by the "European Malaria Vaccine Initiative (EMVI)", at the time hosted by the Statens Serum Institute in Copenhagen. EMVI has by now acquired its own legal personality, such that EMVDA since the end of 2009 is now coordinated by EMVI's successor organization named European Vaccine Initiative (EVI) which is based in Heidelberg.

**Figure 2:**
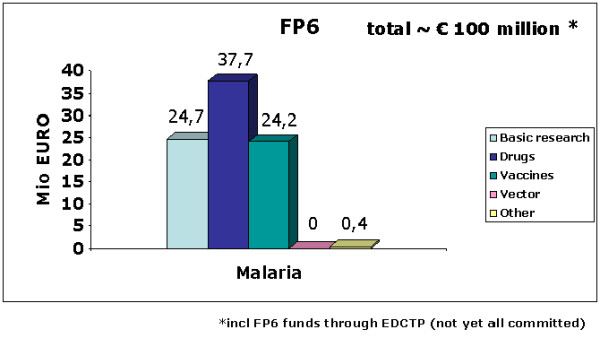
**EU-funded malaria research under FP6 (2002 - 2006), split by thematic subareas**.

Figure 1 also highlights some key features and data on each of the three FP6 cornerstone projects. It should be noted that these three major structural projects were throughout the lifetime of FP6 complemented by a scatter of small scale, innovative malaria projects, and also by other not disease specific research projects looking at cross-cutting issues. The total of EU funding committed to malaria research under FP6, is displayed in Figure 2: approximately € 65 million were directly allocated to malaria projects following FP6 calls, while additional approximately € 35 million (up to 2010), were invested in malaria-relevant intervention trials and research capacities in Africa, supported by EDCTP.

## Continued support for European malaria research under FP7

The on-going 7^th ^EU Framework Programme for research and technological development (2007-2013), again features malaria as an identified target area for research support, as part of research on "Infectious Diseases". Under the first four calls of FP7 thematic sub-areas of malaria research that had not previously been tackled under FP6, were taken up, largely following the FP6 strategy to create large "ERA structuring" malaria projects which can usefully contribute to global research needs in the respective area. Support for malaria basic research is continued under the FP7 Network of Excellence (NoE) EVIMalaR, which aims to further integrate the participating institutions towards forming a virtual "European Malaria Research Institute" [20].

**Figure 3:**
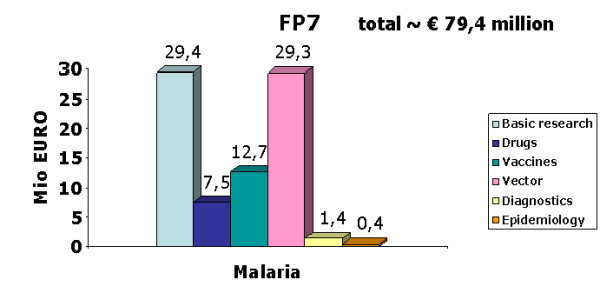
**EU-funded malaria research under FP7 (2007-2013, up to 2010 budget), split by thematic subareas**.

**Figure 4:**
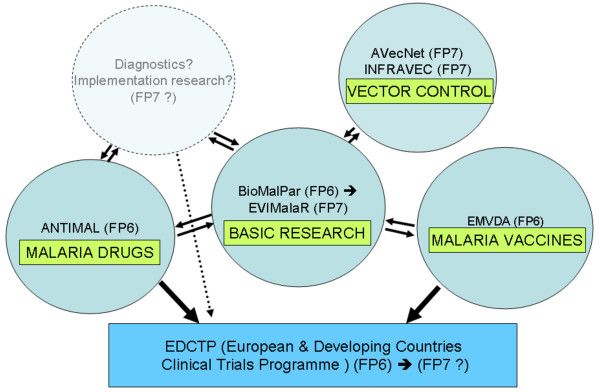
**Large FP6- and FP7-supported malaria projects structuring ERA**.

One major malaria research focus taken up under FP7 concerns research to control malaria transmission by targeting the mosquito vector. An infrastructure project (INFRAVEC) has been established to facilitate genetic modification of the malaria mosquito vector (to make it refractory to the transmission of malaria), and to support rearing and fitness testing of modified mosquitoes in a confined-release facility [21]. This infrastructure effort is complemented by a major transnational research project on new and improved interventions for vector control, called AVecNet, coordinated by Liverpool School of Tropical Medicine, which supports a network of eight European and five African institutions and two European insecticide-producing companies, with EU funding of € 12 million. As yet about € 80 million in total have been committed for malaria research in FP7 (Figure 3) including other areas like pregnancy malaria or basic research topics, that were tackled not only under the FP7 Health research, but also by complementary projects under the FP7 Ideas and People programmes. Future calls under the remaining time of FP7 could address implementation research to evaluate best use of existing and new malaria control interventions in the context of local health systems conditions, including the development and best use of diagnostics (Figure 4).

## Strategic achievements of malaria research under the EU's framework programmes - and lessons learned

Which particular achievements have come out from this, at the time of the start of FP6, new strategic approach, namely to organize European, and African malaria researchers around key topical themes under few large EU-funded project frameworks, that to a good extent are managed by the researchers themselves? Clearly, the administrative burden for the scientists, and in particular for the coordinators, to manage such large EU projects, is significant. Yet, in return, the concept has allowed specific research agendas to be driven by those who are most knowledgeable in the field, i.e. the competent institutions and researchers in the respective fields, with guidance given by independent external expert committees, and by the funding organization, the EU's Directorate-General for Research, who has to ensure that tax payers money is properly spent and accounted for. But there are other assets and advantages that are possible only within the framework of a large strategic umbrella project on the respective subtheme of malaria research: (1) comparability of data sets for several sub-projects e.g. on new drugs or vaccine candidates, allowing for prioritization or de-selection of product candidates, (2) critical mass of research candidates justifies the assembly of comprehensive preclinical assay modules as a central project facility, e.g. comprising harmonized pre-clinical assays and animal models, (3) large EU projects, each involving most of the key European players in the respective thematic areas, can more competently participate in larger international initiatives, (4) within one, and across several, of these larger EU projects, synergies can be exploited e.g. concerning essential horizontal activities, such as joint training schemes. The following examples, extracted from the major FP6- and FP7-funded malaria projects, may more concretely demonstrate these conceptual gains.

Under the FP6-funded malaria drugs project ANTIMAL (Figure 1), 18 sub-projects dealing with different drugs candidates, were grouped under one FP6 "Integrated Project" (IP). While initially all sub-projects received research support, candidate drugs were then continuously scrutinized through comparative evaluation exercises, by an industry-type, competent group of drug developers external to the project, who on the basis of comparable data packages recommended candidate drugs to be further developed, or to be terminated. These recommendations taken up by the project's joint management, thus led to a de-selection of drugs candidates, such that eventually only three of these go into human phase 1 trials. Evidently, this comparative de-selection requires an agreed common project framework, provided through the FP6-funded project structure.

Similarly, the FP6 malaria vaccine project EMVDA has regrouped several European malaria vaccine approaches under one project framework (Figure 1). EMVDA establishes a core module of standardized preclinical assays and models (with standardization still forming part of the project's work programme), in order to allow for comparability of diverse vaccine candidates, before these enter into costly product and clinical development phases. New vaccine candidates were invited to join the project through an open call, however, prior to allocating funds to GMP production and human trials, these new candidates had to pass through EMVDA's agreed preclinical assay package, and produce convincing data to support further investments.

The FP6 malaria basic research network BioMalPar (Figure 1) has, since 2004, developed into a cornerstone of EU malaria research, and has under FP7 been followed by a new network called EVIMalaR. Apart from an impressive output of about 500 original papers, important structural gains resulted from this sustained EU effort in the field of basic research on malaria: the creation of an open annual malaria conference in Europe (BiomalPar conference) that has become a key event in fundamental research in malaria; a strongly integrative European Malaria Graduate School, operated through EMBL in Heidelberg, with central training modules and always linking at least two partner groups on a jointly conducted PhD project; a malaria research protocol book, generated jointly between the American ATCC-based MR4 center and BioMalPar; a popular web-based interactive malaria research teaching tool provided on the BioMalPar website, maintained by the Hebrew University in Jerusalem.

It is noteworthy that all of these projects comprise as full partners a good number of research groups from malaria-endemic countries, mainly from Africa (Figure 1), thus demonstrating the outreach feature of an "Open European Research Area" as called for since the inception of the ERA strategy [13,22]. Other indicators for enhanced visibility and impact of the European malaria consortia at international level: BiomalPar signed a partner agreement with the Australian Malaria Research Network, on joint projects and exchange schemes, financially supported by both the EU Commission's Directorate General for Research and the Australian HHMRC; EVI (coordinating the EMVDA project), alongside EDCTP, participates in the WHO-convened global malaria vaccine funders group and co-funds part of their joint activities; and ANTIMAL has engaged in a global coordination project (CRIMALDDI) where key players in malaria drugs development, including WHO/TDR, MMV and ANDi, (the African network for Drugs and Diagnostics Innovation) intend to align research efforts for a more rationally organized and accelerated development of new anti-malarial drugs.

The strategic and international impact generated by these large EU-funded malaria research consortia is to some extent contrasted by sometimes complex administrative procedures that have to be applied to the management of projects funded with public monies, for example when implementing dynamic elements like competitive calls by which additional partners join an ongoing project. Future development of funding tools should take into account the need for dynamic changes during the lifetime of a project.

## Conclusions

A new strategic concept, brought forward at the start of FP6, to "build ERA" in the field or malaria, was introduced, tested and has shown to bear fruit. Highly active EU-funded key consortia filled the concept with life and built dedicated European malaria research initiatives that have become key stakeholders in their respective fields, both in the European and in the global context. In the case of EU-funded malaria research, a powerful and timely cocktail of a number of favourable factors promoted enhanced impact of European malaria research: (1) supportive political stimuli, (2) responsive programming, (3) clear definition of the thematic area, and (4) readiness of the targeted research community. While the general concept to structure the European Research Area (ERA) through large, strategic FP6 and FP7 projects proved generally successful in terms of generating impact and visibility, funding tools may be developed further in the spirit of general simplification of programme management, to provide for more dynamics and flexibility in the conduct of large strategic project efforts.

## Abbreviations

ANDi: African Network for Drugs and Diagnostics Innovation; ATCC: American Type Culture Collection; BMGF: Bill and Melinda Gates Foundation; EDCTP: European and Developing Countries Clinical Trials Partnership; EMBL: European Molecular Biology Laboratory; EMVI: European Malaria Vaccine Initiative; ERA: European Research Area; EU: European Union; EVI: European Vaccine Initiative; FP: Framework Programme for research, technological development and demonstration activities; GDP: Gross Domestic product; GMAP: Global Malaria Action Plan; GMP: Good Manufacturing Practice; HIV/AIDS: Human Immunodeficiency Virus/Acquired Immunodeficiency Syndrome; INCO: International Co-operation; IP: Integrated Project; MR4: Malaria Research and Reference Reagent Resource Center; MMV: Medicines for Malaria Venture; MRC: Medical Research Council; NHMRC: National Health and Medical Research Council; NoE: Network of Excellence; WHO: World health Organisation; WHO/TDR: World Health Organisation/Tropical Diseases Research.

## Competing interests

The authors declare that they have no competing interests.

## Authors' contributions

AH was until end 2009 as Scientific Officer in charge of EU-funded malaria research under FP6 and FP7; designed, coordinated and contributed to the writing of the manuscript, MTB is a senior malariologist recently retired from Stockholm University, participated in a number of FP6- and FP7-funded malaria projects; contributed to the writing of the manuscript, IPJ is since beginning of 2010 as Scientific Officer in charge of EU-funded malaria research under FP6 and FP7; contributed to the writing of the manuscript. All the authors reviewed the final manuscript and approved it before submission.
